# Assignment of the CD Cotton Effect to the Chiral Center in Pseurotins, and the Stereochemical Revision of Pseurotin A_2_

**DOI:** 10.3390/md14040074

**Published:** 2016-04-09

**Authors:** Takeshi Yamada, Mina Ohshima, Kaori Yuasa, Takashi Kikuchi, Reiko Tanaka

**Affiliations:** Medicinal Chemistry Laboratory, Osaka University of Pharmaceutical Sciences, 4-20-1, Nasahara, Takatsuki, Osaka 569-1094, Japan; skr-dh.mc-tp.128@ezweb.ne.jp (M.O.); k.m.k.l.v.k@gmail.com (K.Y.); t.kikuchi@gly.oups.ac.jp (T.K.); tanakar@gly.oups.ac.jp (R.T.)

**Keywords:** pseurotins, *Aspergillus fumigatus*, marine microorganism, marine fish, cephalimysins, spiro-heterocyclic γ-lactam

## Abstract

Pseurotins A_1_ (**1**) and A_2_ (**2**) were isolated from a culture broth of the fungal strain *Aspergillus fumigatus* WFZ-25 as stereoisomers of pseurotin A (**3**) in 2011. We also isolated **1** and **2** together with **3** from *A. fumigatus* OUPS-T106B-5 separated from the marine fish *Mugil cephalus*. In this study, we re-examined the stereochemistry of **1** and **2** using chemical transformation and the CD spectra, and found the relationship between the CD Cotton effect and the absolute configurations of **1** and **2**, which led us to revise the stereostructure of pseurotin A_2_.

## 1. Introduction

Pseurotin A (**3**) is a major secondary metabolite isolated from the fungal strains *Pseudeurotium ovalis* and *Aspergillus fumigatus*, and it has an unusual structure containing a spiro-heterocyclic γ-lactam core [1,2,3,4]. Its absolute configuration was determined by X-ray diffraction analysis of a dibromo derivative [1]. Most of the other γ-lactams were determined by asymmetric total synthesis [5,6,7,8,9,10] and the modified Mosher’s method [11,12]. Previously, we reported that all stereoisomers of FD-838 showed an association between the CD Cotton effect and the absolute configuration of the chiral centers in γ-lactam [13]. Meanwhile, stereoisomers of pseurotin A designated as pseurotins A_1_ (**1**) and A_2_ (**2**) were isolated from a culture broth of the fungal strain *Aspergillus fumigatus* WFZ-25 by Q.Q. Gu and co-researchers [14]. The absolute stereostructures of **1** and **2** were elucidated by NOESY experiments and comparison with the CD data pattern in the above report [14]. We herein report our re-examination of the absolute configurations of **1** and **2** using chemical transformation, measurement of the ^1^H-NMR coupling constant, and CD spectra. In addition, we describe our revision of the stereochemistry of **2**.

## 2. Results and Discussion

**Figure 1:**
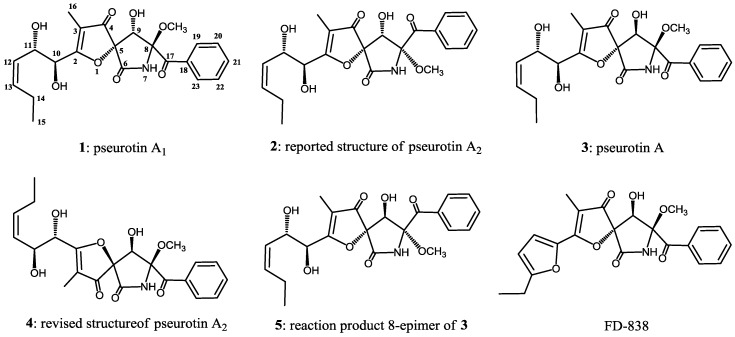
Structures of pseurotins and FD-838.

Fractionation of an ethyl acetate extract of the culture broth of *A. fumigatus* OUPS-T106B-5 was conducted as reported previously [12,13], employing a stepwise combination of Sephadex LH-20 and silica gel column chromatographies, followed by reverse-phase HPLC, to yield pseurotins A_1_ (**1**), A_2_ (**2**) and A (**3**) (Figure 1).

Pseurotin A_1_ (**1**) had the molecular formula C_22_H_25_NO_8_, as established from the [M + Na]^+^ peak in high resolution fast atom bombardment mass spectrometry (HRFABMS). A close inspection of the ^1^H- and ^13^C-NMR spectra of **1** (Table 1, Supplementary Material Figures S1 and S2) using DEPT and ^1^H-^13^C correlation spectroscopy (HMQC) revealed the presence of one primary methyl (C-15), one olefinic methyl (C-16), one methoxy group (8-OCH_3_), one sp^3^-hybridized methylene (C-14), three oxygen-bearing sp^3^-methines (C-9, C-10 and C-11), two olefin sp^2^-methines (C-12 and C-13), two oxygen-bearing quaternary sp^3^-carbons (C-5 and C-8), five aromatic protons (C-19, C-20, C-21, C-22 and C-23), three quaternary sp^2^-carbons (C-2, C-3, C-18) including one oxygen-bearing quaternary carbons (C-2), two conjugated carbonyl groups (C-4 and C-17), one amido (C-6 and N-7) and one hydroxy group (9-OH). The connection of these units was determined on the basis of ^1^H–^1^H COSY and HMBC correlations to reveal the planar structure of **1**, which was identified as being the same as that of pseurotin A_1_ by comparison with data in the literature [14]. In addition, spectroscopic analyses of **2** and **3** identified them as pseurotin A_2_ and pseurotin A, respectively [14] (Supplementary Material Figures S3–S10).

**Figure 2:**
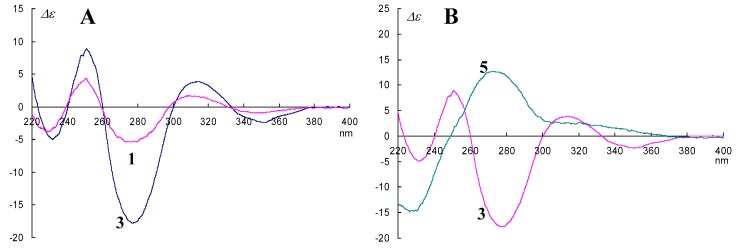
(**A**) The comparison of CD spectra of **1** and **3**; (**B**) The comparison of CD spectra of **3** and **5**.

We succeeded in the isolation of all stereoisomers of FD-838 (Figure 1) including four reaction products, and, therefore, we could establish the relationship between absolute configurations at C-5 and C-8 in the spirofuranone-lactam skeleton and the CD Cotton effects. In addition, we found that the chemical shifts of H-9 and the coupling constant between H-9 and 9-OH in the ^1^H-NMR spectrum with CDCl_3_ as a solvent demonstrated the orientations of 9-OH and 8-OCH_3_ [13]. On investigating the absolute configuration for pseurotin A_1_ (**1**) [14], we applied the above phenomena. Comparing the CD spectral data of **1** and **3**, the similarity of their CD curves showed that the absolute configurations of C-5 and C-8 in **1** were the same as those in **3**, *i.e.*, **1** possessed the 5*S*, 8*S* absolute configuration (Figure 2A). For the absolute configuration at C-9, 9-OH oriented *cis* to 8-OCH_3_ for a large coupling constant (*J* = 12 Hz), and *trans* to 8-OCH_3_ for a small coupling constant (*J* = 4 Hz) in its ^1^H-NMR spectra, and the relative configuration between 9-OH and 8-OCH_3_ regularly influenced the chemical shift of C-9 in its ^1^^3^C-NMR spectra [13]. In this study, we could not observe the coupling constant between H-9 and 9-OH (*vide info*); however, the NMR chemical shifts of C-9 (δ_C_ 76.6) clearly showed that 9-OH oriented *trans* to 8-OCH_3_ [13]. If 9-OH orients *cis* to 8-OCH_3_, the NMR chemical shifts of C-9 would be observed in a high field (δ_C_~74.0) [13]. The above evidence confirmed the absolute stereostructure of **1** [14]. Q.Q. Gu and co-researchers determined the stereochemistry of **1** from NOESY correlations (H-9/8-OCH_3_ and 9-OH/10-OH) and a comparison of the CD data with **3**. In addition, they had referred to our CD spectral examination; however, they had not confirmed the wavelength of the maximum absorbance proceeding from a chiral center of C-8 [13,14].

The extended conjugate system in pseurotins was less marked than those in FD-838 and cephalimysins B–D; therefore, the Cotton effects in the CD spectra should exhibit a hypsochromic shift [13]. In order to assign the Cotton effect ascribed to the configuration at C-8, we examined the epimerization at C-8 in **3**. Treatment of **3** with conc. H_2_SO_4_ in MeOH gave **5**, an 8-epimer of **3**, as reported in the literature [13] (Supplementary Material Figures S11 and S12). The CD spectrum of **5** showed the opposite curve to that of **3** at around 280 nm (Figure 2B), *i.e.*, the negative Cotton effect at around 280 nm demonstrated that the absolute configuration at C-8 was an *S* configuration.

**Figure 3:**
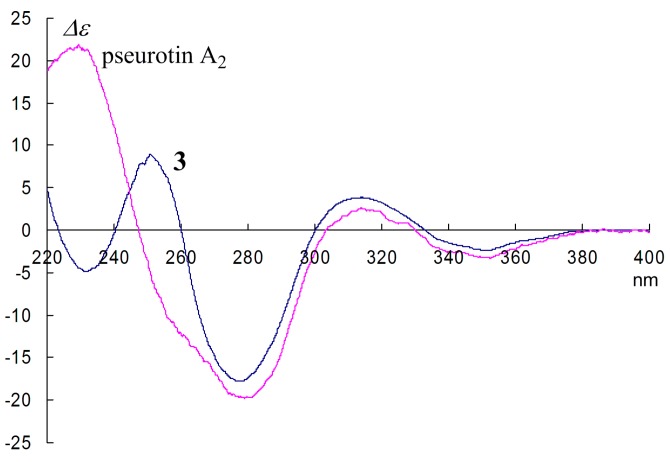
CD spectra of pseurotin A_2_ and **3**.

To confirm the stereostructure of pseurotin A_2_ as **2** [14], a comparison with CD spectral data of pseurotin A_2_ and **3** was carried out. The Cotton effect at around 250 nm in the CD spectral data of pseurotin A_2_ was negative, while that of **3** was positive (Figure 3). Q.Q. Gu *et al.* reported that this difference was due to the change from 8*S* in **3** to 8*R* in **2** [14]; however, the above evidence showed that their deduction should be corrected, *i.e.*, the negative Cotton effect (λ_max_~280 nm) in the CD spectrum of pseurotin A_2_ revealed that the absolute configuration at C-8 was an *S* and not *R* configuration (Figure 3). Meanwhile, the large coupling constant between H-9 and 9-OH in the ^1^H-NMR spectrum (*J* = 12.0 Hz) showed that 9-OH oriented *cis* to 8-OCH_3_, *i.e.*, the absolute configuration at C-9 was an *R* configuration [13]. Q.Q. Gu *et al.* [14] observed a NOESY correlation between 9-OH and 10-OH in **2**, while we could not observe it. This NOESY correlation and the above evidence suggested a reversal of the configuration at C-5 in **2**; therefore, we found that the CD Cotton effect ascribed to the enone moiety (λ_max_~250 nm) could be assigned to the absolute configuration at C-5. Based on the detailed analysis of the CD spectra of pseurotin A_2_ and **3**, the 5*S* isomer **3** showed positive (*λ*_max_~250 nm) and negative (*λ*_max_~230 nm) Cotton effects, while the 5*R* isomer **2**, pseurotin A_2_, showed negative (*λ*_max_~250 nm) and positive (*λ*_max_~230 nm) Cotton effects, respectively (Figure 3). We had demonstrated the same relationship as this phenomenon in our previous report [13], *i.e.*, the 5*S* isomer (FD-838 and cephalimysin B) exhibited a positive Cotton effect, and the 5*R* isomer (cephalimysin C and D) exhibited a negative Cotton effect at around 350 nm, respectively.

**Figure 4:**
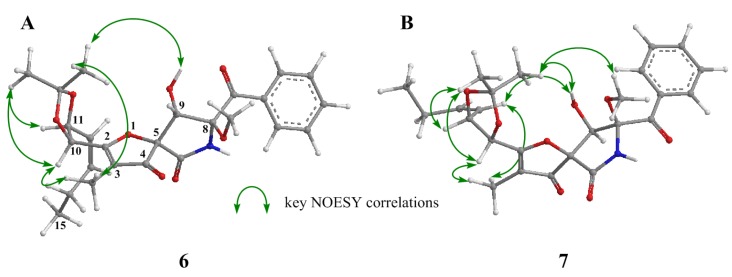
Key NOESY correlations of acetonide derivatives **6** (**A**) and **7** (**B**).

The stereochemistries of C-10 and C-11 in the side chain of pseurotins were not established positively. To build a relative stereochemistry between the spiro γ-lactam moiety and the side chain, we attempted derivatization to acetonide between 10-OH and 11-OH in **1**. The treatment with 2, 2-dimethoxypropane in CH_2_Cl_2_ yielded acetonide **6** (Supplementary Material Figure S13). Its NOESY correlations (acetonide α-CH_3_/9-OH and 3-CH_3_, acetonide β-CH_3_/H-10 and H-11, and H-10/3-CH_3_) clearly showed the absolute conformation of H-10 and H-11 to both be *S* (Figure 4A, Supplementary Material Figure S15). When assuming the stereochemistry in the side chain to be reversed, it was inconsistent with the observed NOESY correlations. Therefore, we deduced that the steric vicinity between the acetonide and 3-CH_3_ restrained the free rotation between C-2 and C-10. The NOESY experiment of acetonide **7** (Supplementary Material Figure S14) derived from **4** by the same procedure gave plenty of information for the elucidation of the absolute stereostructure of **4**, *i.e.*, NOESY correlations (acetonide α-CH_3_/9-OH and 8-OCH_3_, acetonide β-CH_3_/H-10 and H-11, H-10/3-CH_3_, and H-12/3-CH_3_ and 9-OH) were demonstrated in the 10*S*, 11*S* absolute configuration (Figure 4B, Supplementary Material Figure S16). Especially, the correlation between H-12 and 9-OH would not be detected in the 10*R*, 11*R* configuration.

## 3. Experimental Section

### 3.1. General Experimental Procedures

UV spectra were recorded on a Shimadzu (Kyoto, Japan) spectro-photometer U-2000 and IR spectra on a JASCO (Tokyo, Japan) FT/IR-680 Plus. NMR spectra were recorded at 27 °C on Agilent (Santa Clara, CA, USA) NMR-vnmrs600 with tetramethylsilane (TMS) (Nacalai Tesque Inc., Kyoto, Japan) as an internal reference. Mass spectra were determined using a Hitachi M-4000H mass spectrometer. Optical rotatory dispersion (ORD) were recorded on a JASCO J-820 polarimeters. Liquid chromatography over silica gel (mesh 230–400) was performed at a medium pressure. HPLC was run on a JASCO PU-1586 equipped with a differential refractometer (RI-1531) and Cosmosil Packed Column 5C_18_-MSII (25 cm × 20 mm i.d.) (Kyoto, Japan). Analytical TLC was performed on precoated Merck (Darmstadt, Germany) aluminum sheets (DC-Alufolien Kieselgel 60 F254, 0.2 mm) with the solvent system CH_2_Cl_2_–MeOH (19:1), and compounds were viewed under UV lamp and sprayed with 10% H_2_SO_4_ followed by heating.

### 3.2. Fungal Material

A strain of *A. fumigatus* was initially isolated from the marine fish *Mugil cephalus* captured in Katsuura Bay, Japan, in October 2000. The fish was disinfected with EtOH and its gastrointestinal tract applied to the surface of nutrient agar layered in a Petri dish. Serial transfers of one of the resulting colonies provided a pure strain of *A. fumigatus*. The fungal strains were identified by Techno Suruga Laboratory Co., Ltd. (Shizuoka, Japan).

### 3.3. Culturing and Isolation of Metabolites

The fungal strain was cultured at 27 °C for six weeks in a liquid medium (75 L) containing 1% soluble starch and 0.1% casein in 50% artificial seawater adjusted to pH 7.4. The culture was filtered under suction, and the culture filtrate was extracted three times with EtOAc. The combined extracts were evaporated *in vacuo* to afford a mixture of crude metabolites (20.5 g) that exhibited cytotoxicity against the P388 cell line (IC_50_ < 1 μg/mL). The EtOAc extract was passed through a Sephadex LH-20 column using CHCl_3_–MeOH (1:1) as the eluent. The second fraction (13.8 g) was chromatographed on a silica gel column with a hexane–CHCl_3_–MeOH gradient as the eluent to afford Fr. 1 (the 2% MeOH in CHCl_3_ eluate, 3.6 g). Fr. 1 was chromatographed on a silica gel column with a CHCl_3_–MeOH gradient as the eluent to afford Fr. 2 (the 5% MeOH in CHCl_3_ eluate, 1.4 g). Fr. 2 was purified by HPLC using MeOH–H_2_O (70:30) as the eluent to afford Fr. 3 (247.9 mg) and Fr. 4 (45.6 mg). Fr. 3 was purified by HPLC using MeOH–H_2_O (50:50) as the eluent to afford Fr. 5 (168.4 mg). Fr. 5 was purified by ODS HPLC using MeCN–H_2_O (30:70) as the eluent to afford pseurotin A_1_ (**1**, 1.5 mg) and pseurotin A (**3**, 70.5 mg). Fr. 4 was purified by HPLC using MeOH–H_2_O (50: 50) as the eluent to afford Fr. 6 (168.4 mg). Fr. 6 was purified by ODS HPLC using MeCN–H_2_O (30:70) as the eluent to afford pseurotin A_2_ (**4**, 4.1 mg).

Pseurotins A, A_1_ and A_2_: ^1^H- and ^13^C-NMR data (CDCl_3_) are listed in Table 1.

### 3.4. Chemical Transformation

#### 3.4.1. Epimerization of **3**

To a solution of **3** (3.2 mg) in MeOH (0.5 mL), one drop of conc. H_2_SO_4_. was added, and the reaction mixture was stirred at room temperature for 30 min. The reaction mixture was extracted with diethyl ether thrice, and the organic layer was evaporated under reduced pressure. The residue was purified by HPLC using MeCN–H_2_O (30:70) as the eluent to afford **5** (0.6 mg).

#### 3.4.2. Derivatization to Acetonides from Pseurotin A_1_ (**1**) and A_2_ (**4**)

To a solution of **1** (3.3 mg) in CH_2_Cl_2_ (0.3 mL), 2,2-dimethoxypropane (0.3 mL) and pyridium *p*-toluensulfonate (0.2 mg) were added, and the reaction mixture was stirred at room temperature for 1 h. The reaction mixture was evaporated under reduced pressure. The residue was purified by HPLC using MeOH–H_2_O (60:40) as the eluent to afford acetonide **6** (2.2 mg). Using the same procedure, **4** (2.0 mg) was treated with 2,2-dimethoxypropane (0.3 mL) and pyridium *p*-toluensulfonate (0.2 mg), and purified by HPLC to afford **7** (0.4 mg).

Acetonide **6**: Pale yellow oil; FABMS *m*/*z* (rel. int.): HRFABMS *m*/*z* 472.1964 [M + H]^+^ (calcd for C_25_H_30_NO_8_: 472.1970). ^1^H-NMR δ ppm (CDCl_3_): 0.95 (3H, t, *J* = 7.2 Hz, H-15), 1.43 (3H, s, acetonide-β-CH_3_), 1.58 (3H, s, acetonide-α-CH_3_), 1.73 (3H, s, H-16), 2.04 (1H, m, H-14A), 2.17 (1H, m, H-14B), 2.76 (1H, d, *J* = 3.6 Hz, 9-OH), 3.31 (3H, s, 9-OCH_3_), 4.77 (1H, d, *J* = 3.6 Hz, H-9), 5.15 (1H, d, *J* = 7.8 Hz, H-10), 5.25 (1H, ddd, *J* = 9.6, 7.8, 1.2 Hz, H-11), 5.57 (1H, ddt, *J* = 10.8, 9.6, 1.2 Hz, H-12), 5.57 (1H, dtd, *J* = 10.8, 7.2, 1.2 Hz, H-13), 7.33 (1H, br s, H-6), 7.43 (2H, t, *J* = 7.8 Hz, H-20 and H-22), 7.66 (1H, t, *J* = 7.8 Hz, H-21), 8.22 (2H, d, *J* = 7.8 Hz, H-19 and H-23).

Acetonide **7**: Pale yellow oil; FABMS *m*/*z* (rel. int.): HRFABMS *m*/*z* 472.1964 [M + H]^+^ (calcd for C_25_H_30_NO_8_: 472.1970). ^1^H-NMR δ ppm (CDCl_3_): 1.02 (3H, t, *J* = 7.2 Hz, H-15), 1.49 (3H, s, acetonide-β-CH_3_), 1.64 (3H, s, acetonide-α-CH_3_), 1.67 (3H, s, H-16), 2.13 (1H, m, H-14A), 2.20 (1H, m, H-14B), 3.29 (3H, s, 9-OCH_3_), 3.36 (1H, d, *J* = 12.6 Hz, 9-OH), 4.52 (1H, d, *J* = 12.6 Hz, H-9), 5.17 (1H, d, *J* = 6.6 Hz, H-10), 5.26 (1H, ddd, *J* = 9.0, 6.6, 1.2 Hz, H-11), 5.57 (1H, ddt, *J* = 10.8, 9.0, 1.2 Hz, H-12), 5.72 (1H, dtd, *J* = 10.8, 7.2, 0.6 Hz, H-13), 7.49 (2H, t, *J* = 7.8 Hz, H-20 and H-22), 7.64 (1H, t, *J* = 7.8 Hz, H-21), 8.29 (2H, d, *J* = 7.8 Hz, H-19 and H-23).

## 5. Conclusions

Q.Q. Gu *et al.* [14] deduced the stereostructure of pseurotin A_2_ as **2** from NOESY experiments and a comparison of CD spectra with cephalimysins. We assigned the CD Cotton effect ascribed to the absolute configuration at C-8 by the epimerization of pseurotin A (**3**), and revised the stereostructure of pseurotin A_2_ from **2** to **4**. In this process, we newly found the Cotton effect ascribed to the absolute configuration at C-5. In addition, we found that the absolute configuration in the side chain of pseurotins could be established positively by detailed analyses of the NOESY experiments of their acetonide derivatives.

## Figures and Tables

**Table 1:** NMR spectral data forpseurotins in CDCl_3_.

Position	Pseurotin A_1_ (1)				Pseurotin A_2_				3				5			
	δ_H_^a^	m, *J*/Hz	δ_C_		δ_H_^a^	m, *J*/Hz	δ_C_		δ_H_^a^	m, *J*/Hz	δ_C_		δ_H_^a^	m, *J*/Hz	δ_C_	
1																
2			183.4	qC			183.5	qC			186.0	qC			186.4,	qC
3			113.2	qC			114.3	qC			113.4	qC			114.8,	qC
4			196.2	qC			199.7	qC			196.5	qC			201.1,	qC
5			89.5	qC			87.3	qC			92.7	qC			86.5,	qC
6			169.4	qC			166.9	qC			166.8	qC			167.5,	qC
7	8.53	s			7.70	s			8.38	s			7.34	s		
8			96.5	qC			93.2	qC			90.5	qC			96.4,	qC
9	4.88	s	76.6	CH	4.42	br d, 12.0 (9-OH)	74.2	CH	4.69	br s	73.2	CH	4.86	d, 5.4 (9-OH)	78.6,	CH
10	4.60	br s	70.5	CH	4.73	d, 3.0 (11)	70.1	CH	4.59	d, 5.4 (11)	70.7	CH	4.69	br d, 5.4 (11)	70.9,	CH
11	4.76	d, 7.8 (12)	71.0	CH	4.94	dd, 8.4 (12) 3.0 (10)	70.6	CH	4.75	dd, 10.8 (12) 5.4 (10)	70.7	CH	4.81	dd, 10.8 (12) 5.4 (10)	69.6,	CH
12	5.23	dd, 10.8 (13) 7.8 (11)	126.4	CH	5.28	dd, 10.8 (13) 8.4 (11)	125.3	CH	5.28	dd, 11.2 (13) 10.8 (11)	126.4	CH	5.43	dd, 11.2 (13) 10.8 (11)	125.6,	CH
13	5.64	dt, 10.8 (12) 7.2 (14)	136.9	CH	5.64	dt, 10.8 (12) 7.2 (14)	137.4	CH	5.59	dt, 11.2 (12) 7.8 (14)	136.8	CH	5.74	dt, 11.2 (12) 7.8 (14)	138.2,	CH
14A	2.09	m	21.4	CH_2_	2.14	m	21.4	CH_2_	2.09	m	21.4	CH_2_	2.15	m	21.5,	CH_2_
14B	2.15	m			2.19	m			2.15	m			2.21	m		
15	0.99	t, 7.8 (14)	14.1	CH_3_	1.03	t, 7.2 (14)	14.1	CH_3_	0.98	t, 9.0 (14)	14.1	CH_3_	1.05	t, 7.2 (14)	14.2,	CH_3_
16	1.68	s	6.2	CH_3_	1.67	s	5.9	CH_3_	1.68	s	6.0	CH_3_	1.78	s	5.6,	CH_3_
17			194.3	qC			194.0	qC			195.2	qC			192.4,	qC
18			133.5	qC			132.8	qC			132.4	qC			133.8,	qC
19	8.27	d, 8.4 (20)	130.0	CH	8.34	d, 8.4 (20)	130.7	CH	8.31	d, 8.4 (20)	130.7	CH	8.20	d, 8.4 (20)	129.4,	CH
20	7.49	t, 8.4 (19, 21)	128.8	CH	7.48	t, 8.4 (19, 21)	128.6	CH	7.49	t, 8.4 (19, 21)	128.7	CH	7.50	t, 8.4 (19, 21)	128.8,	CH
21	7.64	t, 8.4 (20, 22)	134.4	CH	7.63	t, 8.4 (20, 22)	134.6	CH	7.64	t, 8.4 (20, 22)	134.7	CH	7.63	t, 8.4 (20, 22)	134.2,	CH
22	7.49	t, 8.4 (21, 23)	128.8	CH	7.48	t, 8.4 (21, 23)	128.6	CH	7.49	t, 8.4 (21, 23)	128.7	CH	7.50	t, 8.4 (21, 23)	128.8,	CH
23	8.27	d, 8.4 (22)	130.0	CH	8.34	d, 8.4 (22)	130.7	CH	8.31	d, 8.4 (22)	130.7	CH	8.20	d, 8.4 (22)	129.4,	CH
8-OCH_3_	3.37	s	51.7	CH_3_	3.30	s	51.9	CH_3_	3.44	s	51.8	CH_3_	3.27	s	51.5,	CH_3_
9-OH	3.94	br s			4.22	br d, 12.0 (9)			4.25	br s			4.97	d, 5.4 (9)		

^a^ ^1^H chemical shift values (δ ppm from SiMe_4_) followed by multiplicity and then the coupling constants (*J*/Hz). Figures in parentheses indicate the proton coupling with that position.

## Supplementary Materials

The following are available online at www.mdpi.com/1660-3397/14/4/74/s1, Figure S1: ^1^H NMR spectrum in CDCl_3_ of pseurotin A_1_ (**1**), Figure S2: ^13^C NMR spectrum in CDCl_3_ of pseurotin A_1_ (**1**), Figure S3: ^1^H NMR spectrum in CDCl_3_ of pseurotin A_2_ (**4**), Figure S4: ^13^C NMR spectrum in CDCl_3_ of pseurotin A_2_ (**4**), Figure S5: 2D NMR spectra of pseurotin A_2_ (**4**) (^1^H-^1^H COSY), Figure S6: 2D NMR spectra of pseurotin A_2_ (**4**) (HMQC), Figure S7: 2D NMR spectra of pseurotin A_2_ (**4**) (HMBC), Figure S8: ^1^H NMR spectrum in CDCl_3_ of pseurotin A (**3**), Figure S9: ^13^C NMR spectrum in CDCl_3_ of pseurotin A (**3**), Figure S10: ^1^H NMR spectrum in CDCl_3_ of pseurotin A (**3**) (400 MHz), Figure S11: ^1^H NMR spectrum in CDCl_3_ of **5** (8-epimer of **3**), Figure S12: ^13^C NMR spectrum in CDCl_3_ of **5** (8-epimer of **3**), Figure S13: ^1^H NMR spectrum in CDCl_3_ of **6** (acetonide of **1**), Figure S14: ^1^H NMR spectrum in CDCl_3_ of **7** (acetonide of **4**), Figure S15: NOESY of **6** (acetonide of **1**), Figure S16: NOESY of **7** (acetonide of **4**).
