# Supplementary material for: Assignment of the CD Cotton Effect to the Chiral Center in Pseurotins, and the Stereochemical Revision of Pseurotin A2
Source: Mar Drugs. 2016 Apr 9;14(4):74. doi: 10.3390/md14040074 (PMC4849078; doi:10.3390/md14040074)
Supplement: Supplementary file 1 [file marinedrugs-14-00074-s001.pdf]

Supplementary Materials: Assignment of the CD Cotton Effect to the Chiral Center in Pseurotins, and the Stereochemical Revision of Pseurotin A$_2$

Takeshi Yamada, Mina Ohshima, Kaori Yuasa, Takashi Kikuchi and Reiko Tanaka

Figure S1. ^{1}H NMR spectrum in $CDCl_3$ of pseurotin A$_1$ (**1**) S2
Figure S2. ^{13}C NMR spectrum in $CDCl_3$ of pseurotin A$_1$ (**1**) S3
Figure S3. ^{1}H NMR spectrum in $CDCl_3$ of pseurotin A$_2$ (**4**) S4
Figure S4. ^{13}C NMR spectrum in $CDCl_3$ of pseurotin A$_2$ (**4**) S5
Figure S5. 2D NMR spectra of pseurotin A$_2$ (**4**) (^{1}H–^{1}H COSY) S6
Figure S6. 2D NMR spectra of pseurotin A$_2$ (**4**) (HMQC) S7
Figure S7. 2D NMR spectra of pseurotin A$_2$ (**4**) (HMBC) S8
Figure S8. ^{1}H NMR spectrum in $CDCl_3$ of pseurotin A (**3**) S9
Figure S9. ^{13}C NMR spectrum in $CDCl_3$ of pseurotin A (**3**) S10
Figure S10. ^{1}H NMR spectrum in $CDCl_3$ of pseurotin A (**3**) (400 MHz) S11
Figure S11. ^{1}H NMR spectrum in $CDCl_3$ of **5** (8-epimer of **3**) S12
Figure S12. ^{13}C NMR spectrum in $CDCl_3$ of **5** (8-epimer of **3**) S13
Figure S13. ^{1}H NMR spectrum in $CDCl_3$ of **6** (acetonide of **1**) S14
Figure S14. ^{1}H NMR spectrum in $CDCl_3$ of **7** (acetonide of **4**) S15
Figure S15. NOESY of **6** (acetonide of **1**) S16
Figure S16. NOESY of **7** (acetonide of **4**) S17

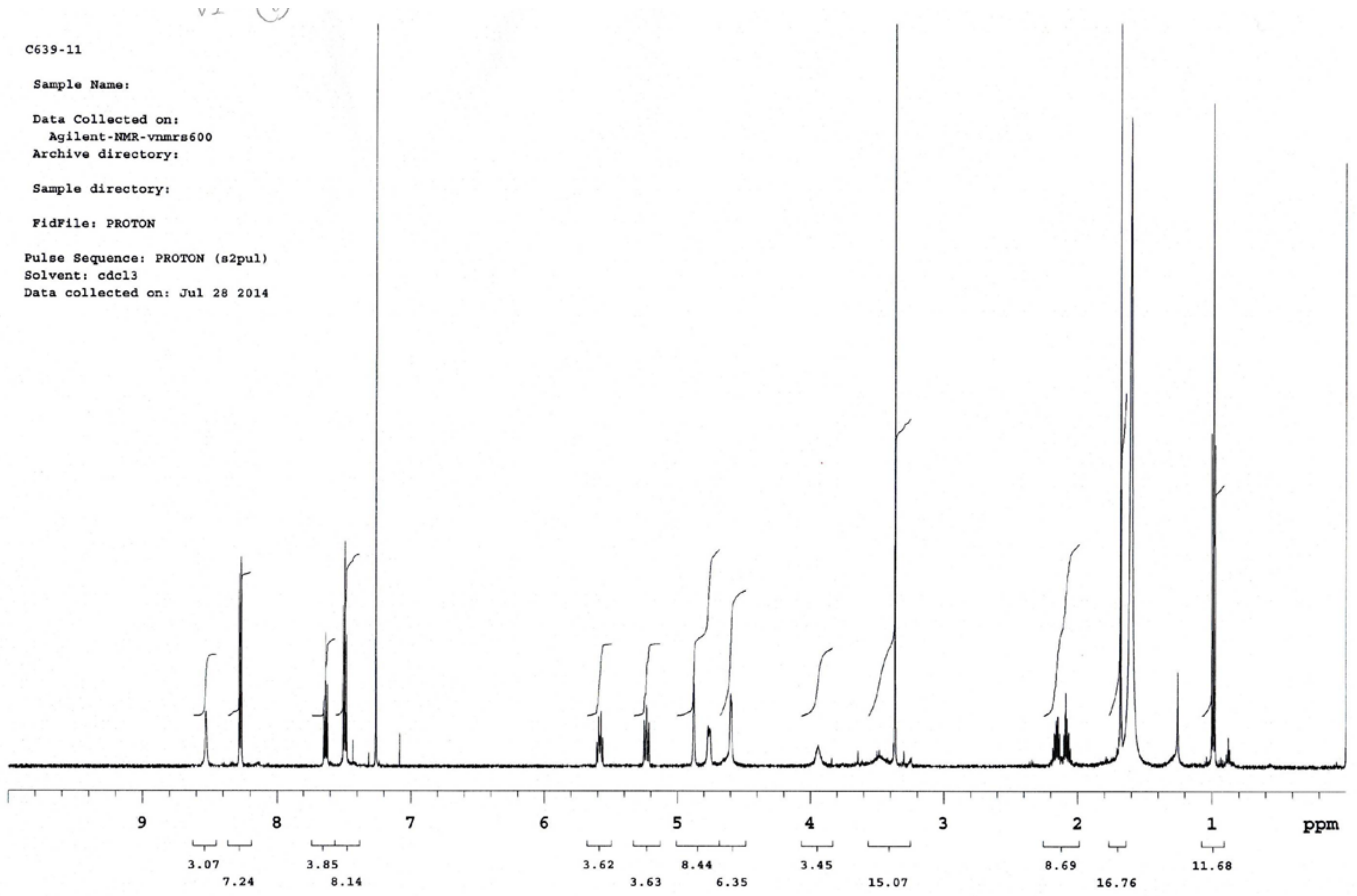

Figure S1. ^{1}H NMR spectrum in $CDCl_3$ of pseurotin A_1 (**1**).

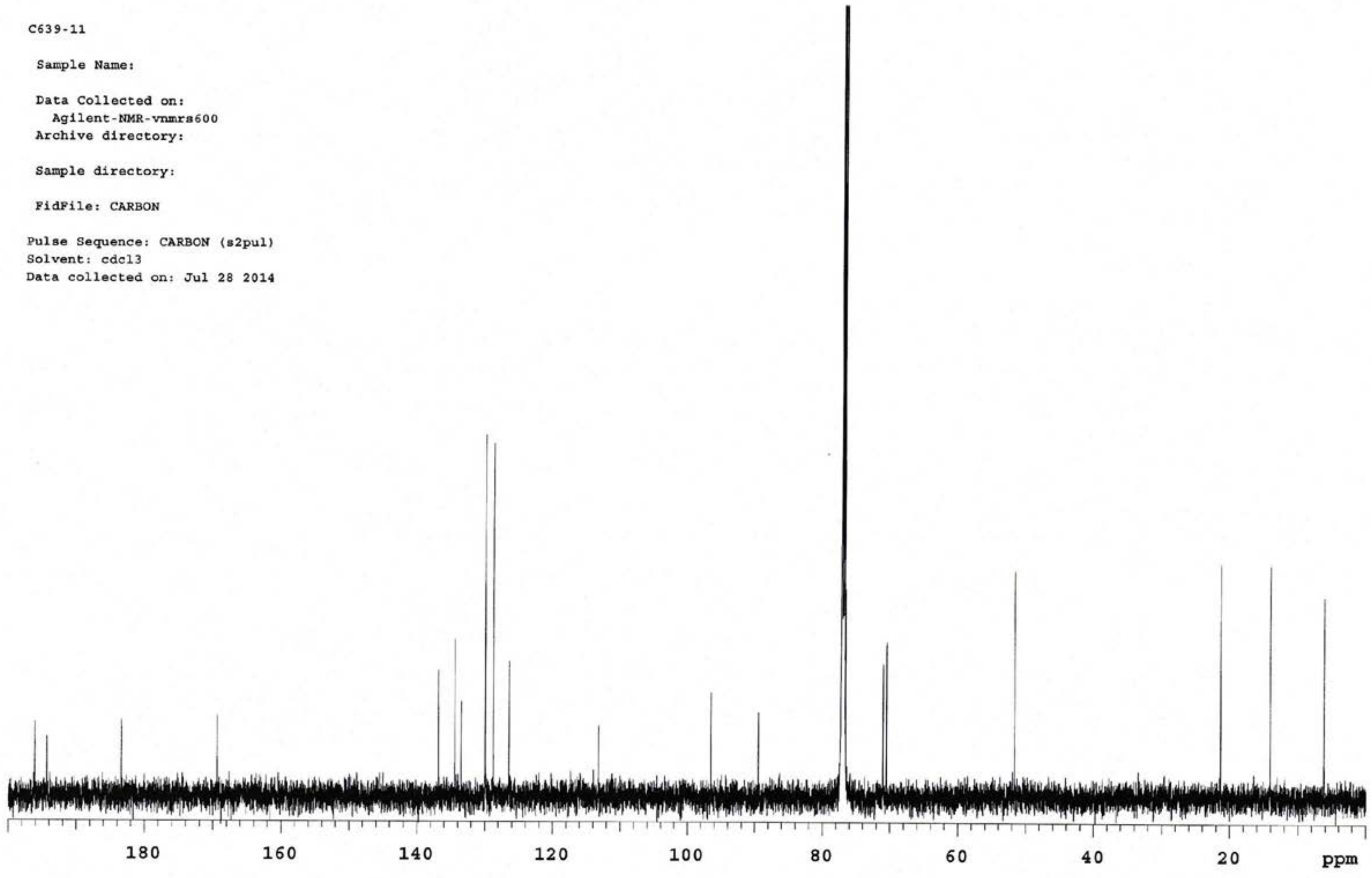

Figure S2. ^{13}C **NMR** spectrum in $CDCl_3$ of pseurotin A_1 (**1**).

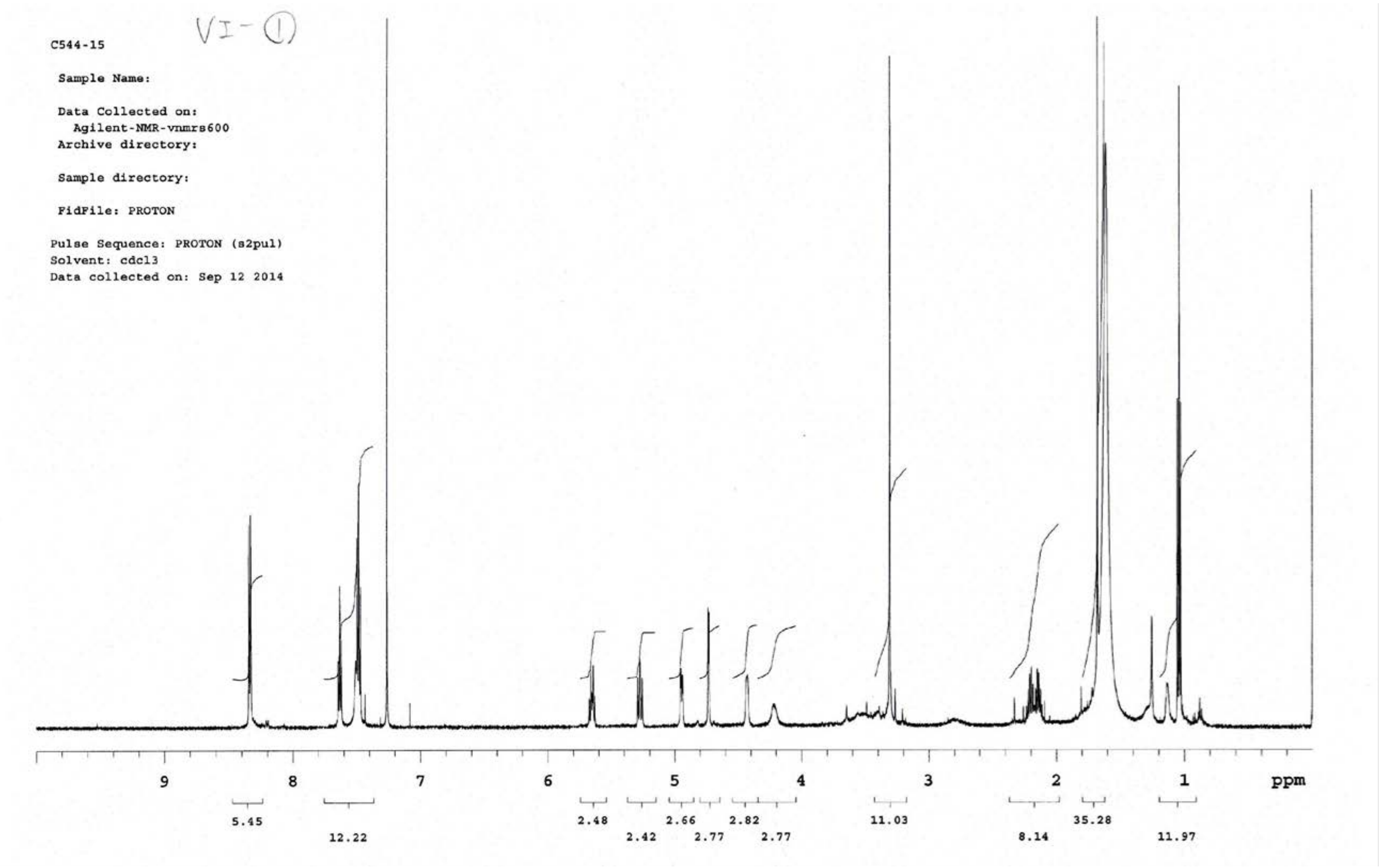

Figure S3. ^{1}H NMR spectrum in $CDCl_3$ of pseurotin A_2 (**4**).

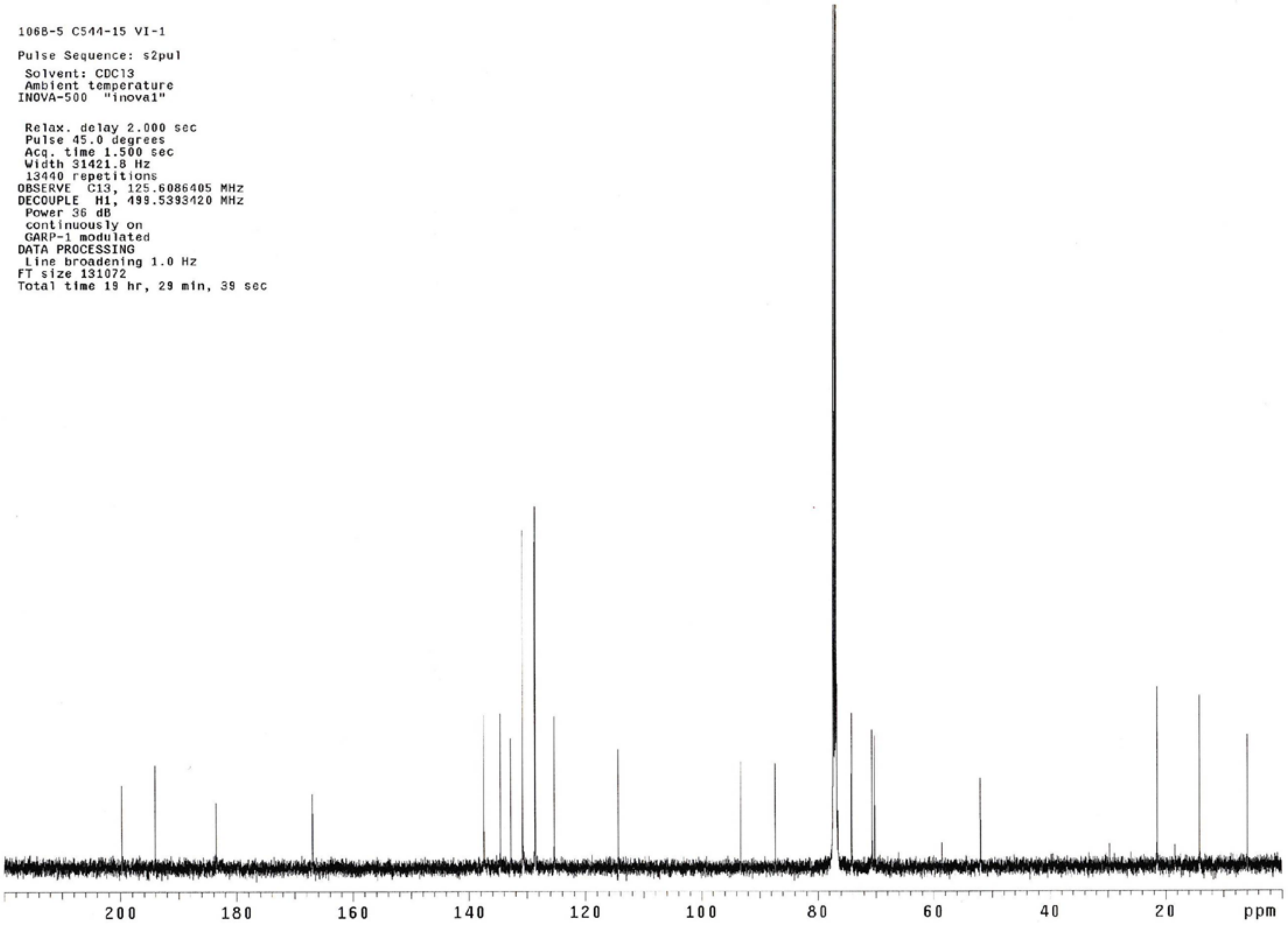

Figure S4. 13C NMR spectrum in $CDCl_3$ of pseurotin A_2 (**4**).

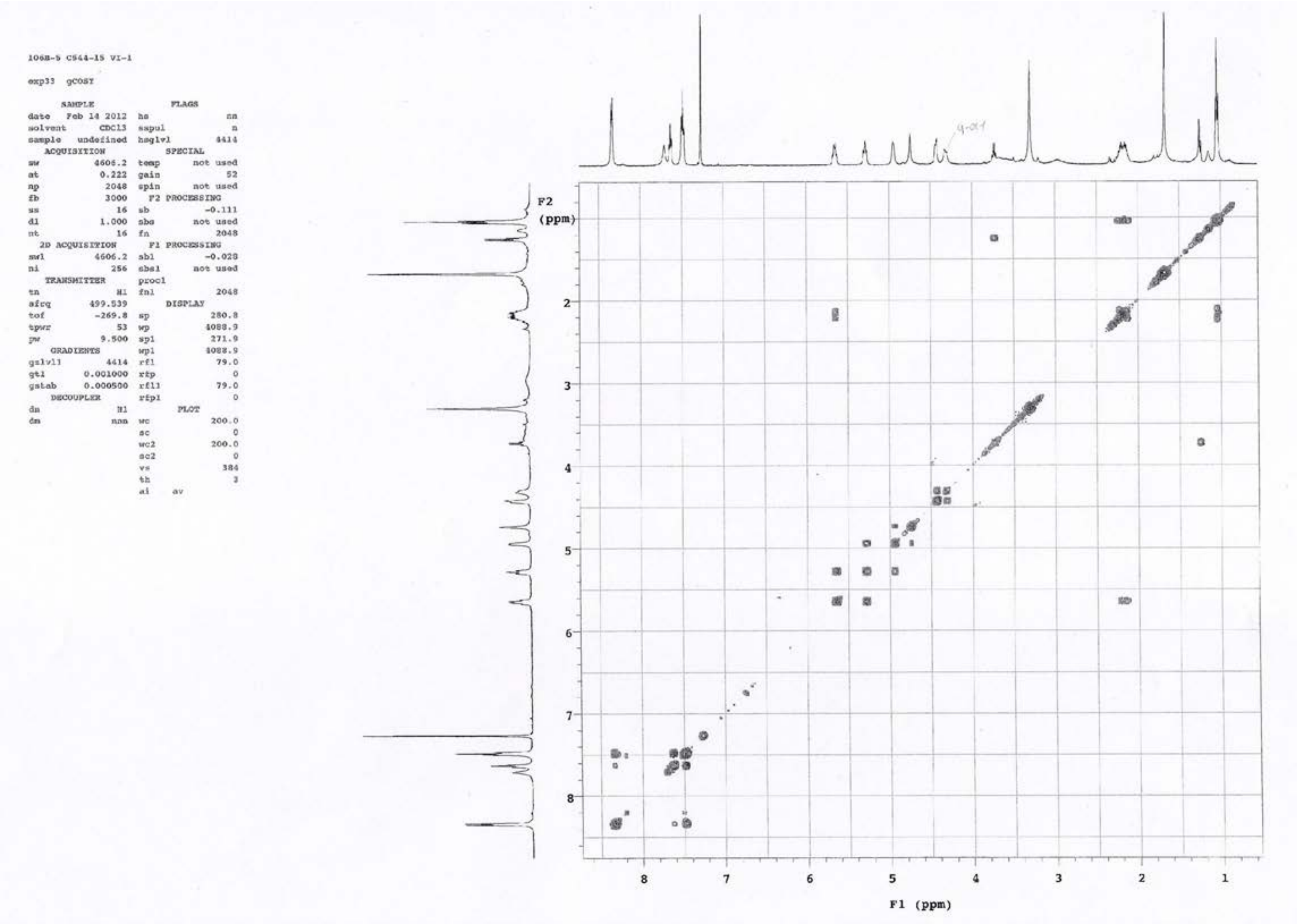

Figure S5. 2D NMR spectra of pseurotin A2 (**4**) (^{1}H–^{1}H COSY).

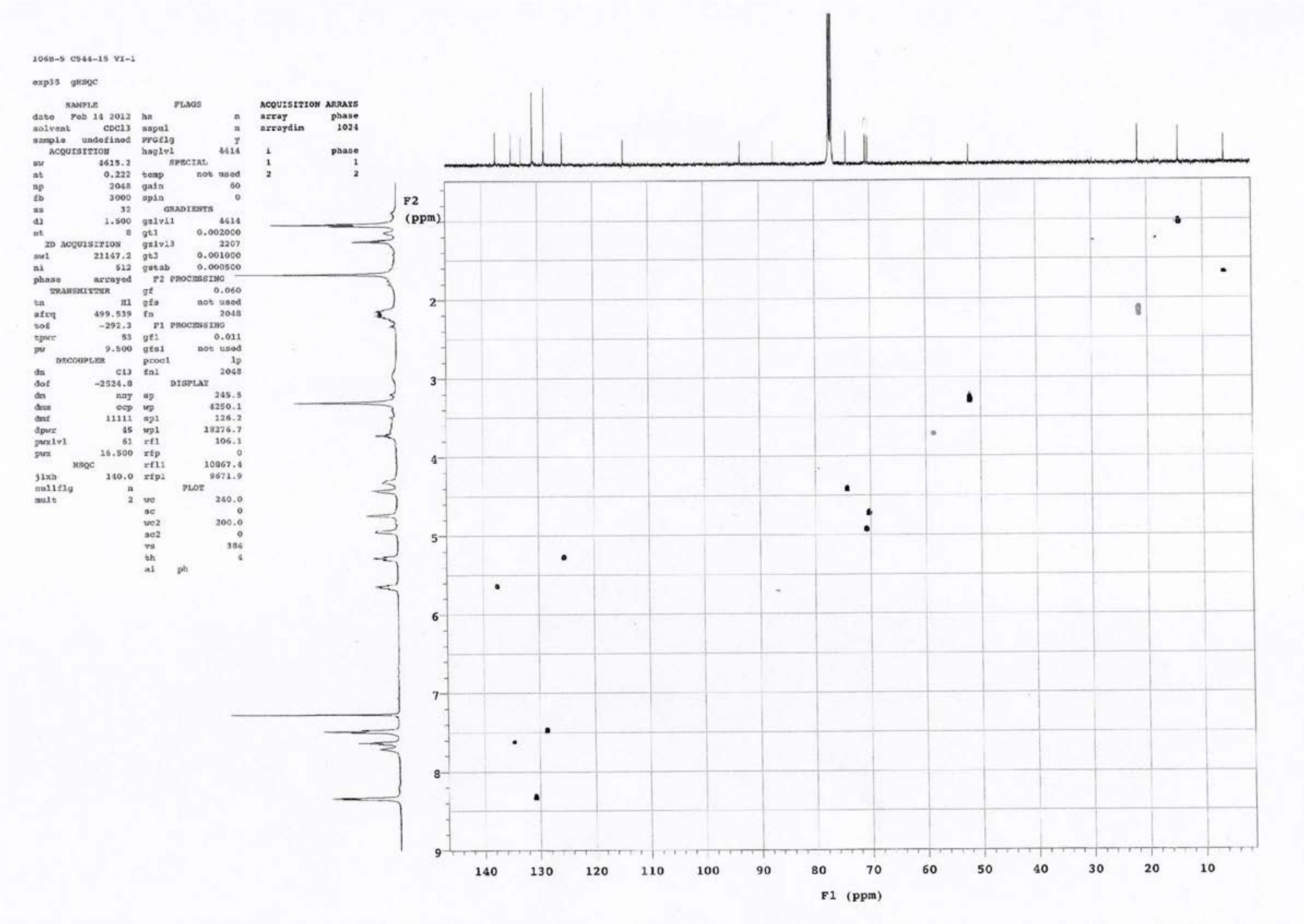

Figure S6. 2D NMR spectra of pseurotin A_2 (**4**) (HMQC).

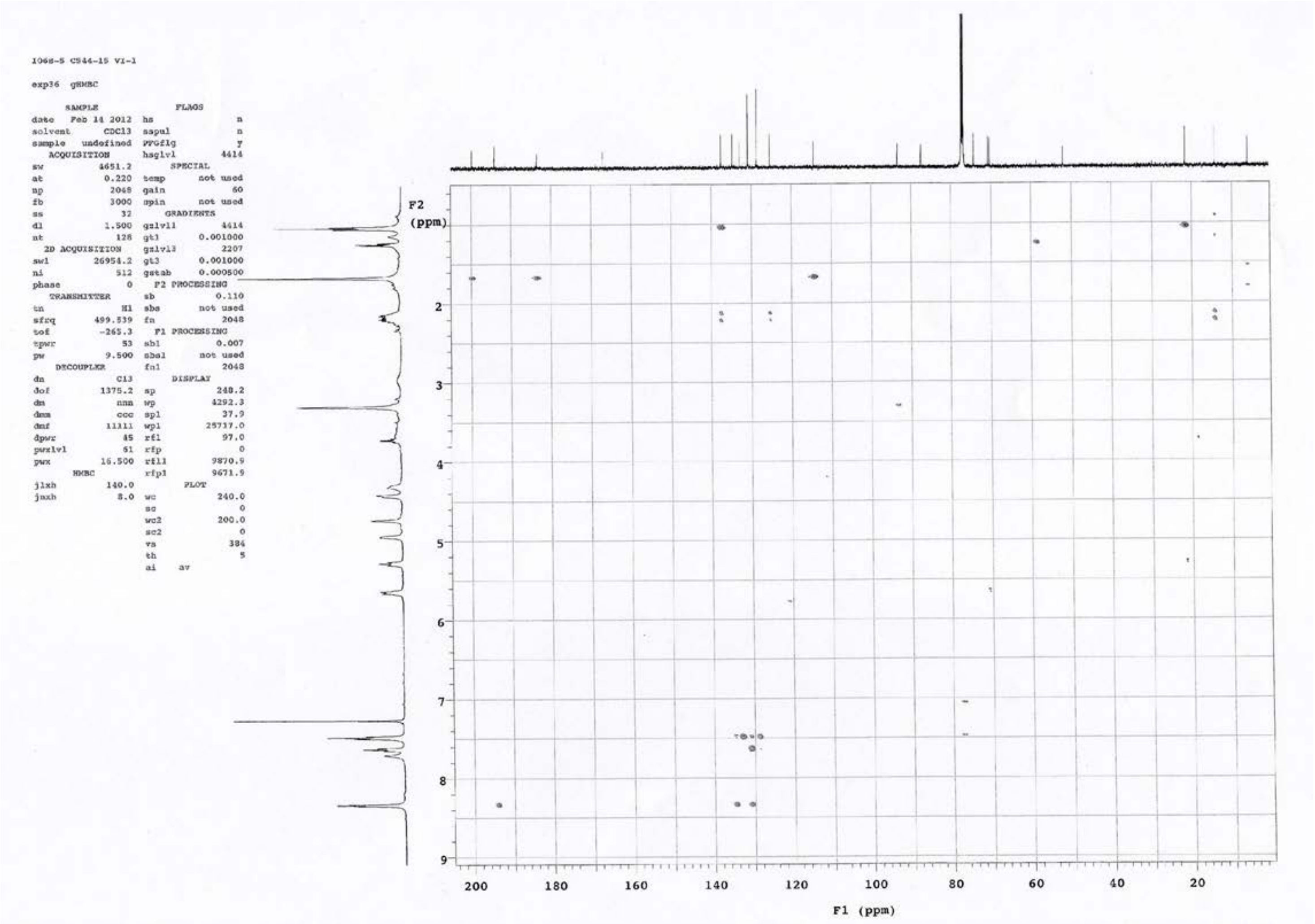

Figure S7. 2D NMR spectra of pseurotin A2 (**4**) (HMBC).

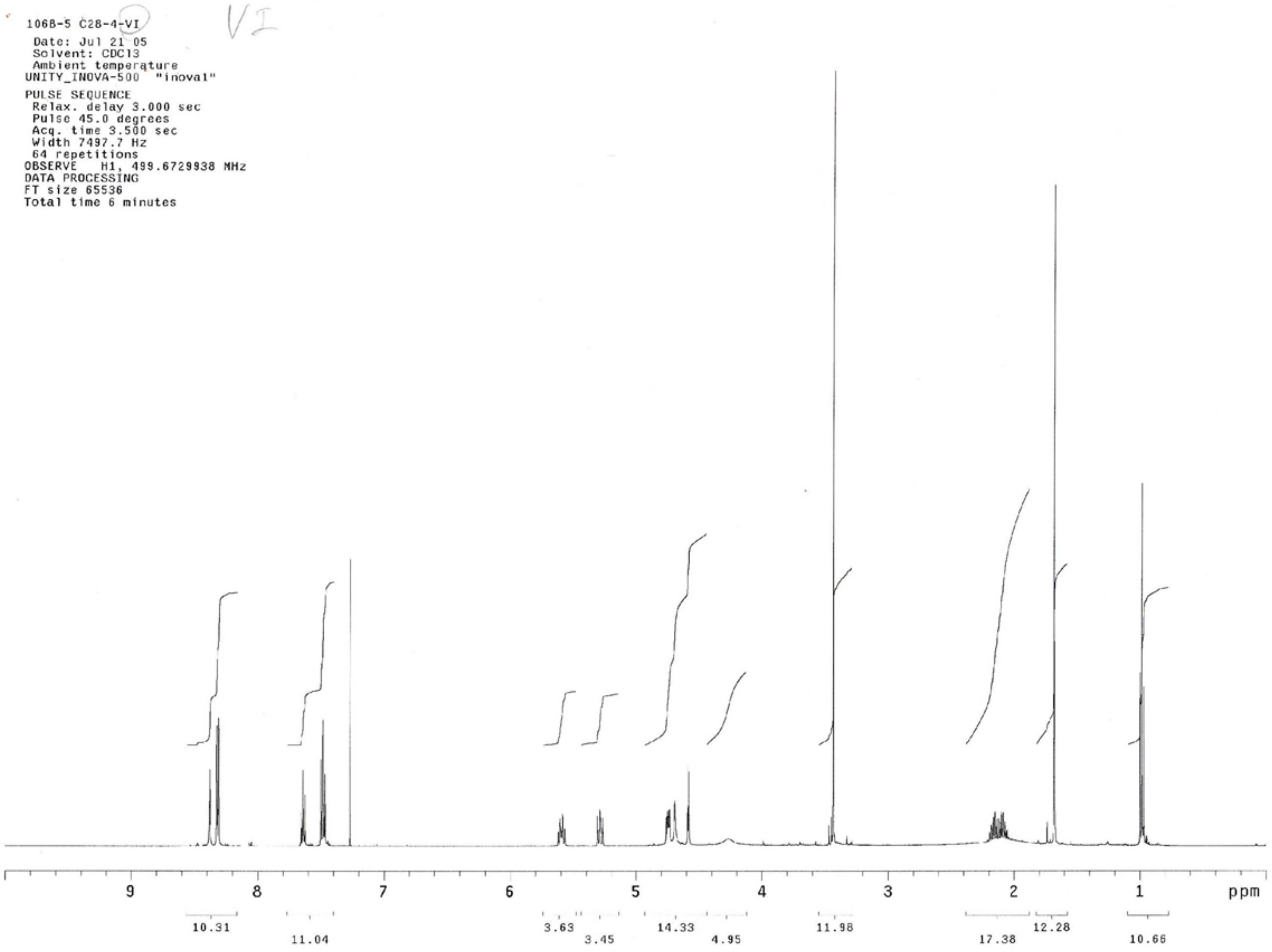

Figure S8. ^{1}H NMR spectrum in $CDCl_3$ of pseurotin A (**3**).

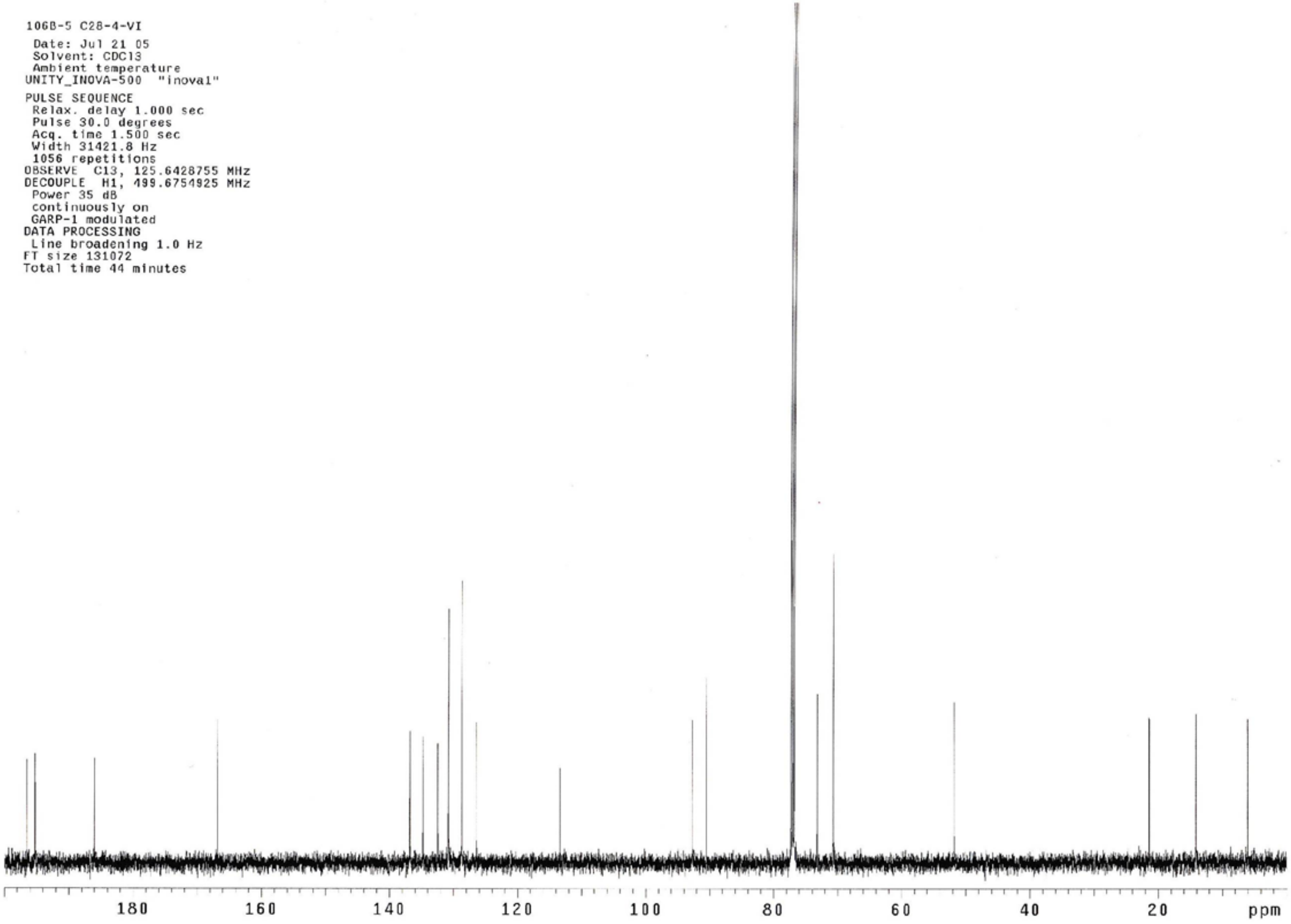

Figure S9. 13C NMR spectrum in $CDCl_3$ of pseurotin A (**3**).

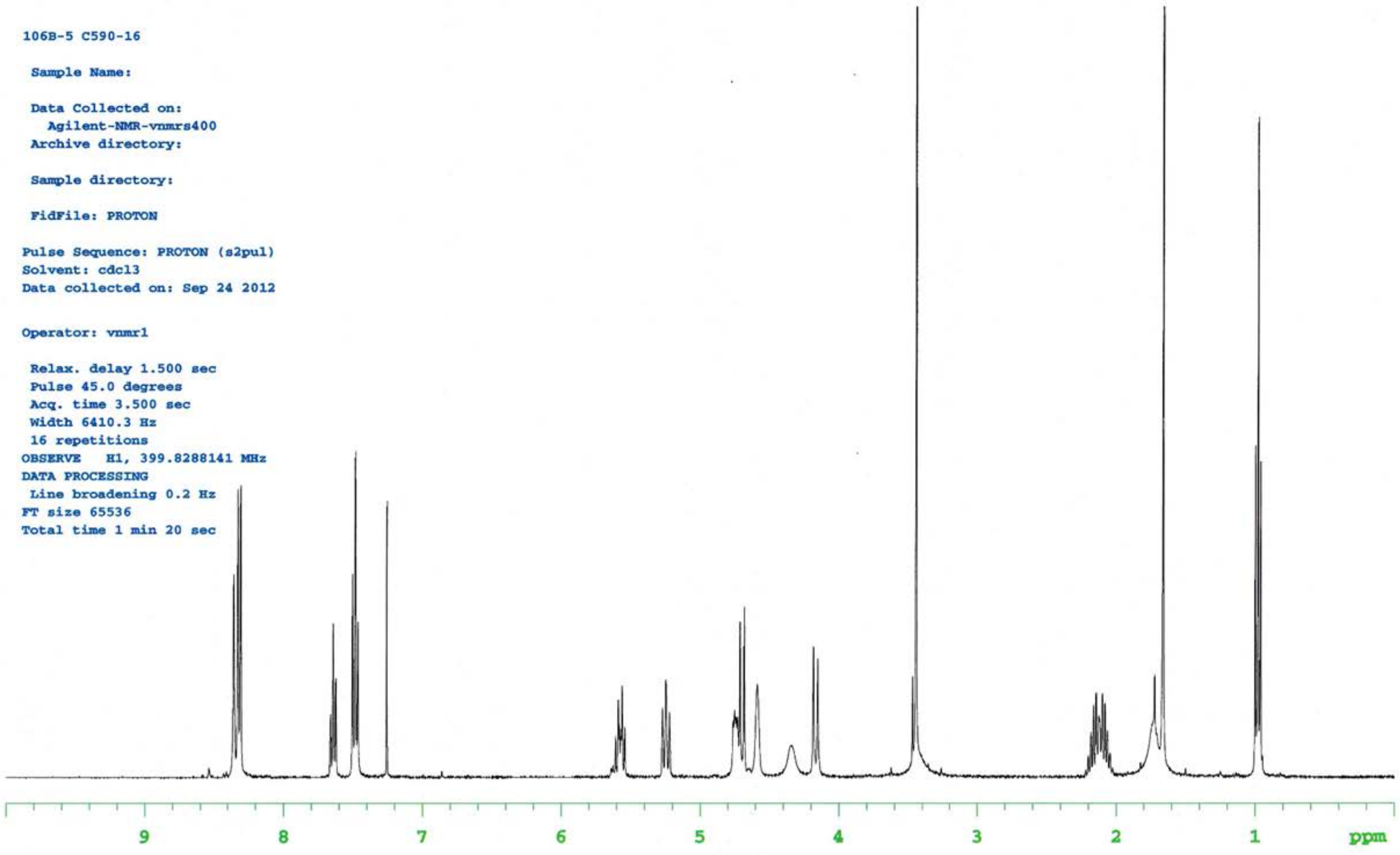

Figure S10. ^{1}H NMR spectrum in $CDCl_3$ of pseurotin A (**3**) (400 MHz).

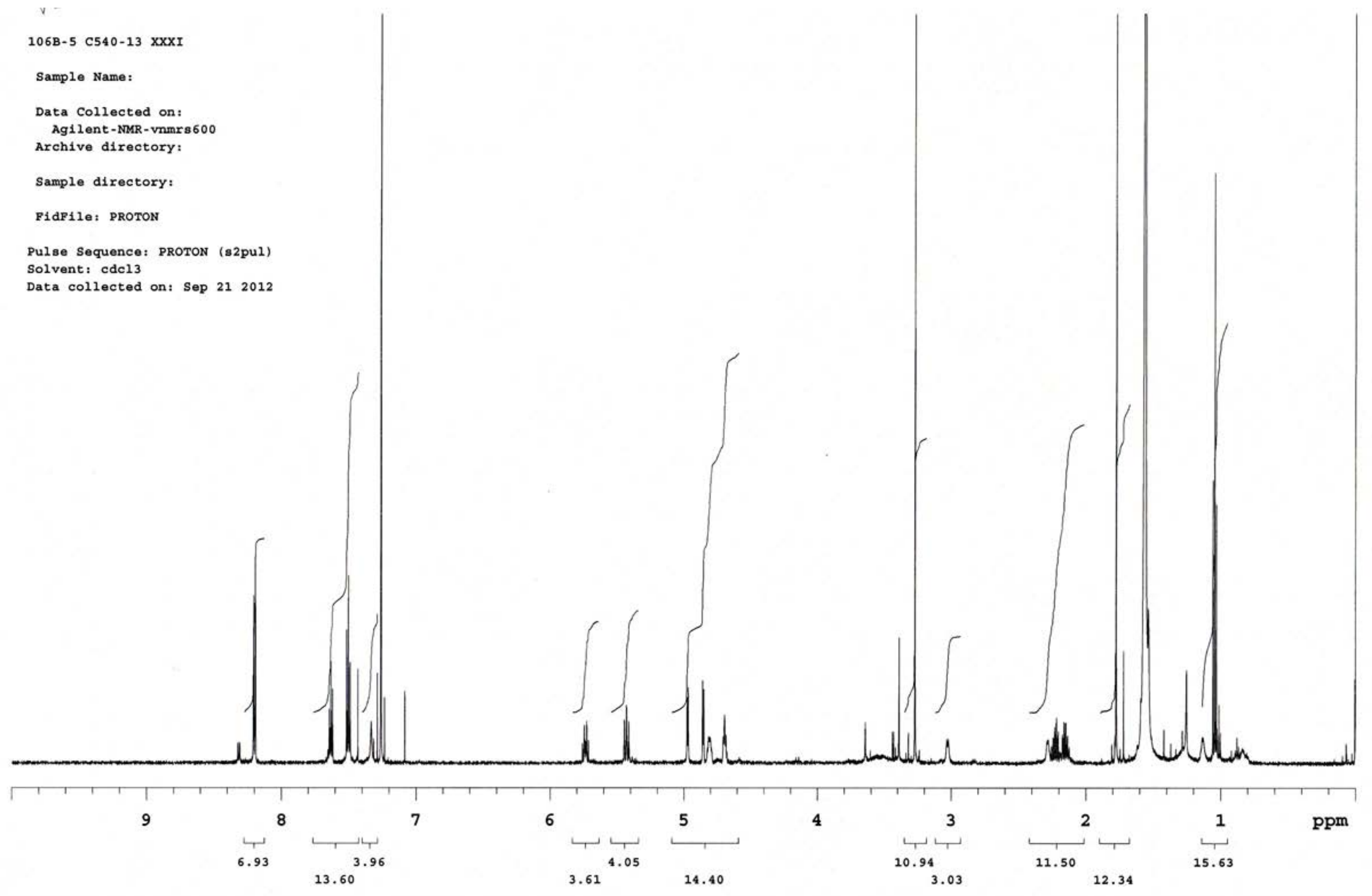

Figure S11. ^{1}H NMR spectrum in $CDCl_3$ of **5** (8-epimer of **3**).

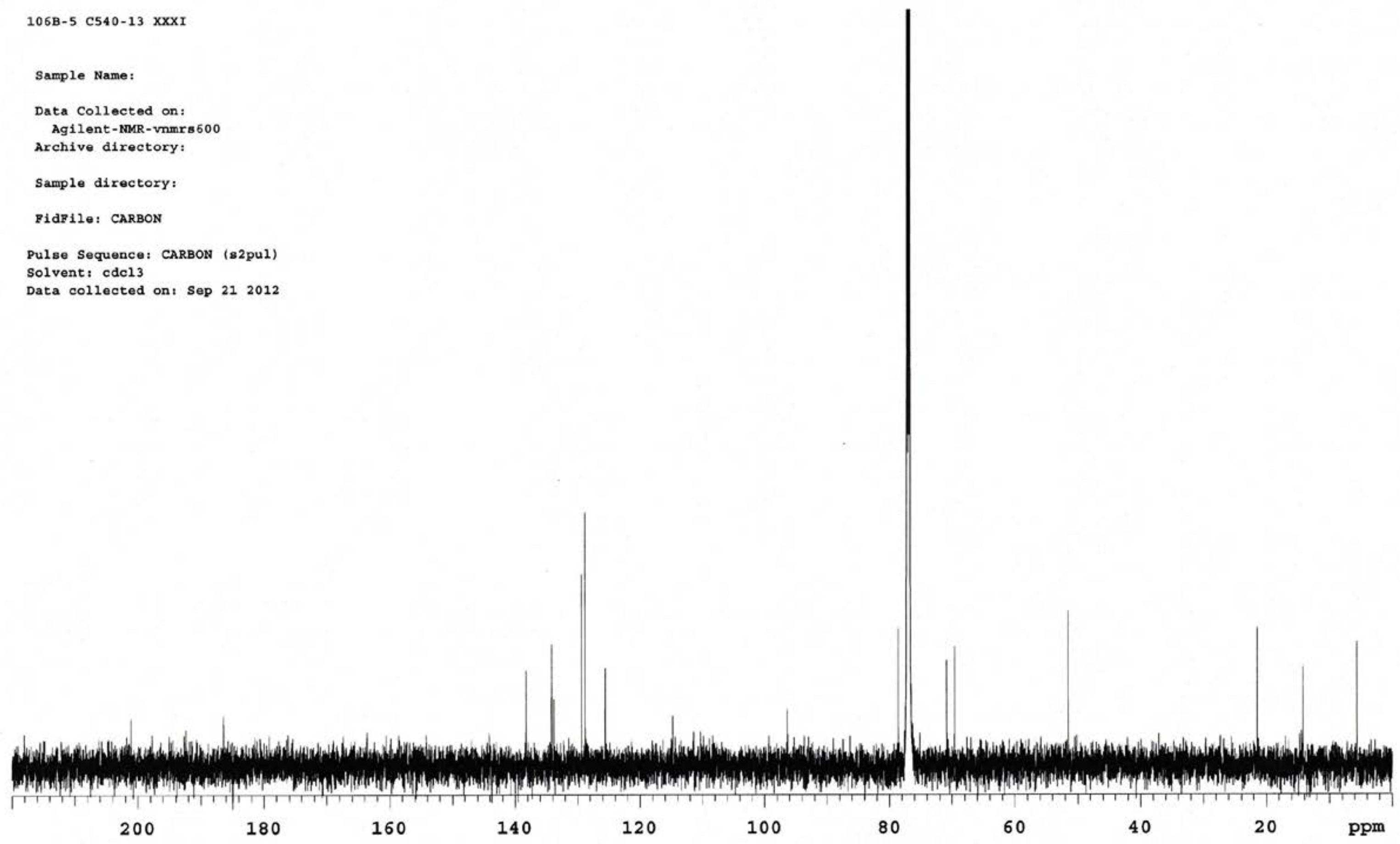

Figure S12. ^{13}C NMR spectrum in $CDCl_3$ of **5** (8-epimer of **3**).

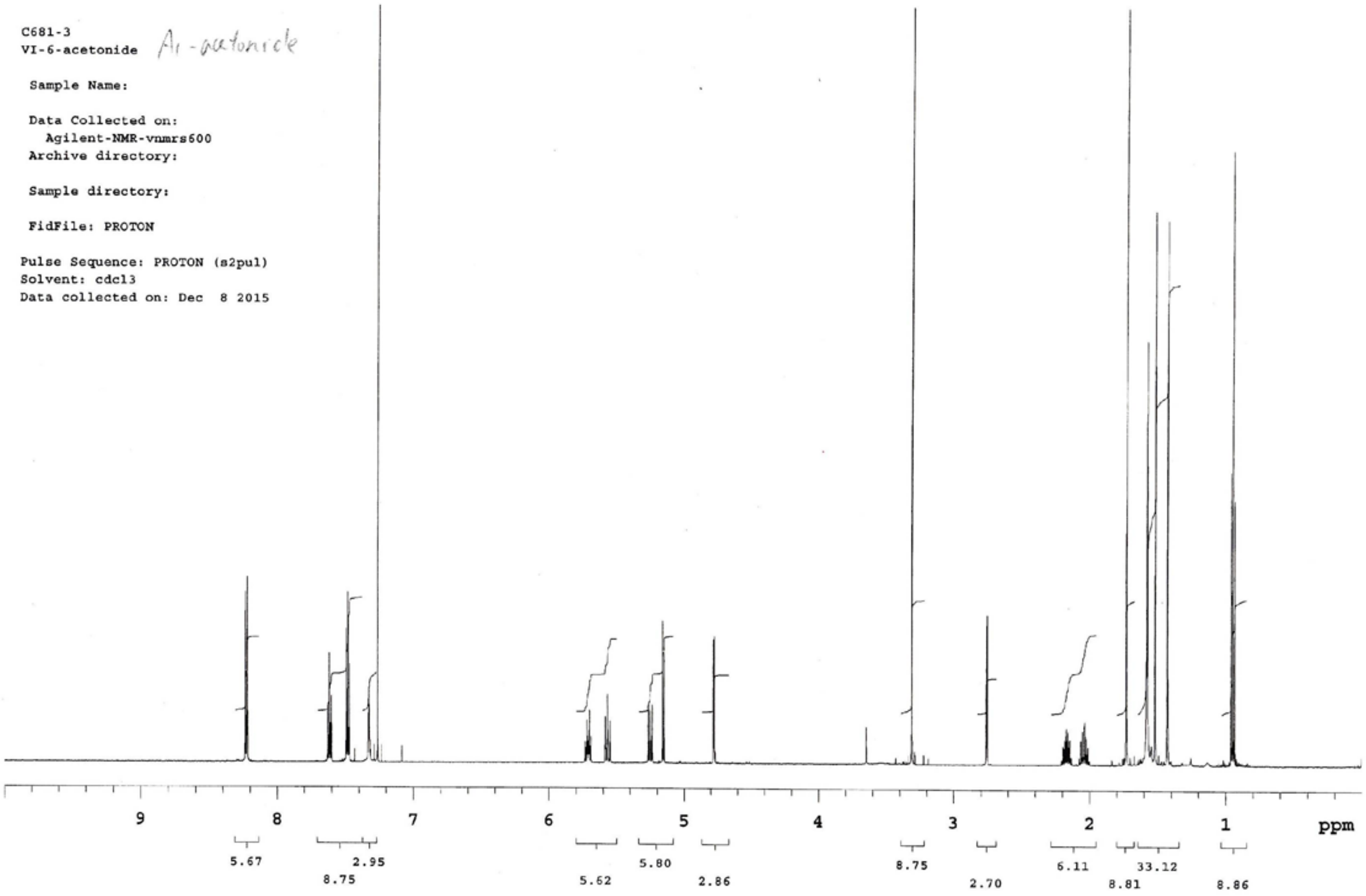

Figure S13. ^{1}H NMR spectrum in $CDCl_3$ of **6** (acetonide of **1**).

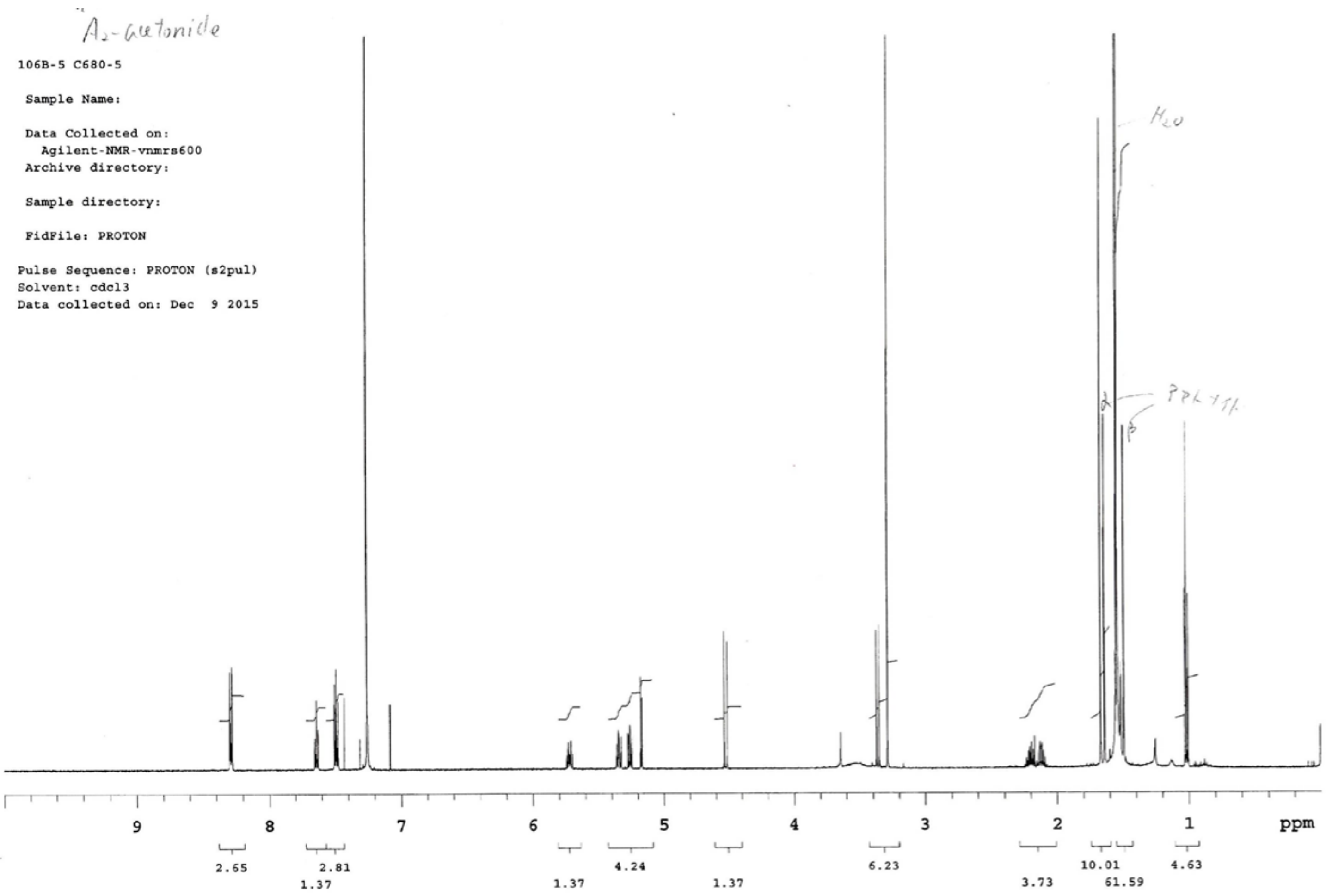

Figure S14. ^{1}H NMR spectrum in $CDCl_3$ of **7** (acetonide of **4**).

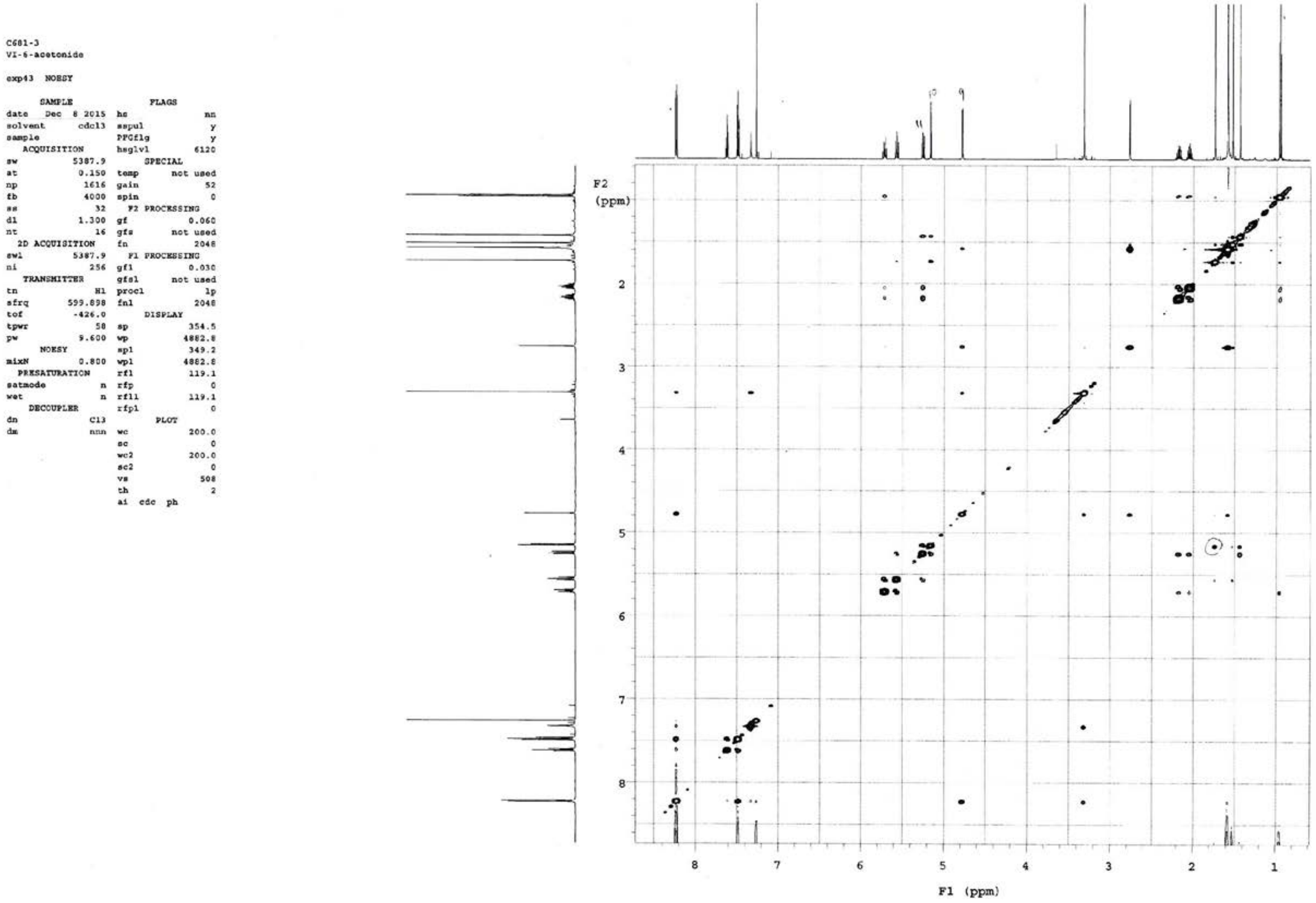

Figure S15. NOESY of **6** (acetonide of **1**).

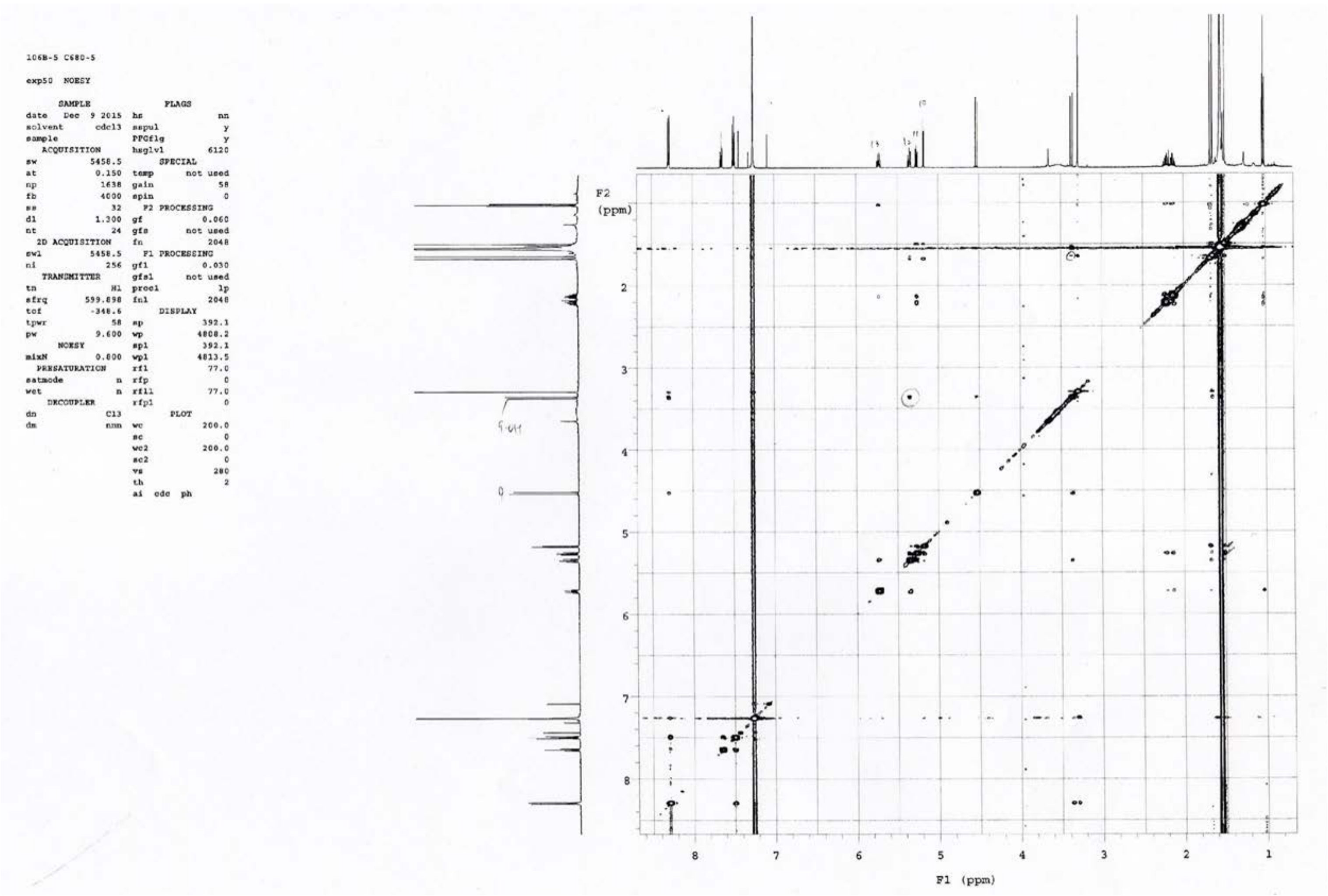

Figure S16. NOESY of **7** (acetonide of **4**).
